# Understanding the Photodynamic Therapy Induced Bystander and Abscopal Effects: A Review

**DOI:** 10.3390/antiox12071434

**Published:** 2023-07-16

**Authors:** Kave Moloudi, Paromita Sarbadhikary, Heidi Abrahamse, Blassan P. George

**Affiliations:** Laser Research Centre, Faculty of Health Sciences, Doornfontein Campus, University of Johannesburg, Johannesburg 2028, South Africa; 223138872@student.uj.ac.za (K.M.); paromitas@uj.ac.za (P.S.); habrahamse@uj.ac.za (H.A.)

**Keywords:** abscopal effect, bystander effect, cancer, calcium signaling, immune response, photodynamic therapy, reactive oxygen species, reactive nitrogen species, T cell

## Abstract

Photodynamic therapy (PDT) is a clinically approved minimally/non-invasive treatment modality that has been used to treat various conditions, including cancer. The bystander and abscopal effects are two well-documented significant reactions involved in imparting long-term systemic effects in the field of radiobiology. The PDT-induced generation of reactive oxygen and nitrogen species and immune responses is majorly involved in eliciting the bystander and abscopal effects. However, the results in this regard are unsatisfactory and unpredictable due to several poorly elucidated underlying mechanisms and other factors such as the type of cancer being treated, the irradiation dose applied, the treatment regimen employed, and many others. Therefore, in this review, we attempted to summarize the current knowledge regarding the non-targeted effects of PDT. The review is based on research published in the Web of Science, PubMed, Wiley Online Library, and Google Scholar databases up to June 2023. We have highlighted the current challenges and prospects in relation to obtaining clinically relevant robust, reproducible, and long-lasting antitumor effects, which may offer a clinically viable treatment against tumor recurrence and metastasis. The effectiveness of both targeted and untargeted PDT responses and their outcomes in clinics could be improved with more research in this area.

## 1. Introduction

Photodynamic therapy (PDT) has been clinically approved as a relatively safe and effective treatment option for certain types of cancer as well as noncancerous skin conditions such as psoriasis, acne, and infections. In principle, PDT is a two-stage procedure whereby a (i) photosensitizer (PS), i.e., a drug that is sensitive to light, is excited by light of an appropriate wavelength, resulting in (ii) the production of cytotoxic reactive oxygen species (ROS) that destroy abnormal cells. PDT has drawn more attention as a therapeutic approach, both as an independent antitumor therapy and as an immunogenic cell death (ICD) approach, due to new mechanistic insights. Until recently, it was widely believed that the mechanisms of action of PDT were mediated by the direct intracellular generation of ROS, which induces a plethora of direct cellular effects such as abnormal cell death, tumor vasculature damage, and the activation of both innate and adaptive immune responses against tumor masses [1]. However, several recent observations have shown that non-irradiated cells can occasionally exhibit the same responses as irradiated cells, whether they are close by (the bystander effect) or located far away (the abscopal effect), thereby disproving this assumption. For many years, the bystander and abscopal effects of ionizing radiation therapy have been well documented and well described. With more mechanistic insights and clinical studies, other non-conventional local and nonionizing radiation therapies like PDT have also been shown to induce the bystander and abscopal effects. However, there is a great deal that is still unknown regarding issues like the nature of signaling, different types of reactions, and the mediator molecules involved [2,3,4].

In brief, the bystander and abscopal responses are regarded as non-targeted effects, which vary from a direct treatment effect. The extent of these effects in terms of the distance from the original site of treatment varies with studies and heavily depends on the interaction of irradiated and unirradiated cells. The bystander effects describe the process whereby the naive (bystander) cells that experience certain biological effects transmitted from proximal cells are directly subjected to therapeutic interventions mediated by gap junctional intercellular communication, the diffusion of soluble factors (including nitric oxide (NO) and ROS), and oxidative metabolism [5,6]. The abscopal effect in cancer therapy, meaning “away from the target site”, is a local-therapy-mediated systemic effect that has the potential to cause distant, non-treated tumor lesions to regress and reject, constituting a process that is suggested to be heavily induced by antitumor immune responses [7,8]. The biological effects of these phenomena are important for the treatment of cancer and preventing tumor recurrence, but they can also affect healthy normal cells. Unfortunately, these effects, particularly the abscopal effect, are still considered to “come by chance, not through seeking”. However, in the past decade, growing interest in this research area and evidence from clinical trials are providing proof that the chances of enhancing an abscopal response can be improved using several different treatment strategies [9].

In this review, we present an overview of the evidence of PDT that have demonstrated adequate responses in light of the resurgence of interest in nontargeted cancer responses like bystander and abscopal effects during the era of advanced and personalized cancer therapies. Though the pathways involved in these two effects are not extensively discussed herein, a general framework of the mechanisms involved in these two phenomena with respect to PDT is covered in detail.

## 2. PDT-Mediated Anticancer Effects

PDT in oncology involves three main components, namely, a PS, light, and oxygen, whose combination selectively destroys cancer cells [10,11]. In clinical settings, the administration of PSs intravenously or through a topical or oral route (used to a lesser extent) results in their preferential accumulation in neoplastic cells, which is followed by light irradiation via a specific light beam focused precisely on the target lesion, which facilitates a second level of selectivity. The selective excitation of the PSs in the neoplastic cells and tumor mass provokes a series of photophysical and photochemical reactions with the surrounding biomolecules in the presence of molecular oxygen, generating ROS such as singlet oxygen and free radicals. The generated cytotoxic ROS cause damage to cellular components such as proteins, lipids, and DNA, leading to cancer cell death via apoptosis or necrosis, which are ultimately required for the treatment of cancer [12,13,14]. In Table 1, three generations of PS drugs have been listed.

The PDT-mediated anticancer effect is in turn mediated by a cascade of complex mechanisms involving (i) direct tumor destruction mediated by ROS generation within the cells induced via the light excitation of a PS; (ii) tumor vasculature damage, leading to the restriction of oxygen and nutrient supply; and (iii) an antitumor immune response, which occurs in response to signals or molecules released due to PDT-induced tumor damage, thus further potentiating the innate and adaptive immunity through activating the complement cascade, the release of pro-inflammatory cytokines, and the fast migration of neutrophils, macrophages, and dendritic cells into the tumor mass, resulting in the elimination of any remaining tumor cells [15]. PDT-induced tumor cell death occurs either through (1) conventional cell death such as apoptosis, necrosis, or autophagy [16] or (2) via nonconventional or new forms of cell death, including mitotic catastrophe (MC), paraptosis, pyroptosis, parthanatos [17], necroptosis, and ferroptosis [16,18] (Table 1). 

**Figure 1:**
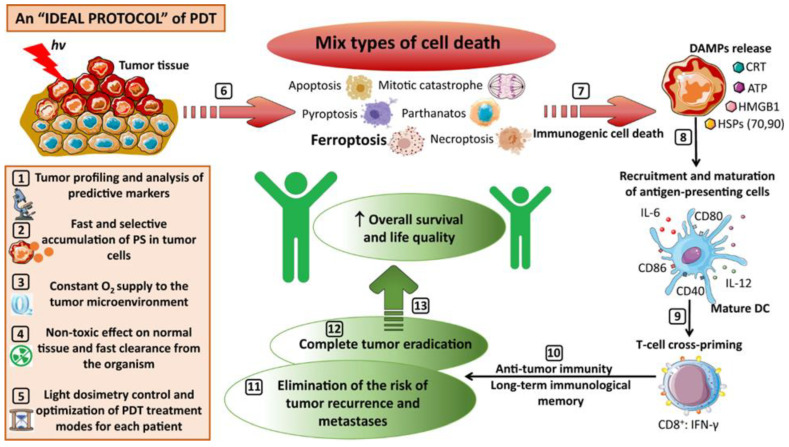
A schematic representation of an ideal protocol for clinical PDT. Adapted with permission from Mishchenko et al. [16], Copyright 2022 SPRINGER NATURE.

The mechanism by which PDT causes cell death has a significant impact on the immune response that develops after treatment, which may improve a treatment’s antitumor effectiveness. According to most of the relevant concepts in this regard, the overall inflammatory response brought on by PDT-induced necrosis is primarily responsible for immune cell recruitment to the tumor area and total tumor eradication. This idea of enhanced antitumor PDT activity is strengthened by the fact that the macrophages that respond only attack necrotic tumor cells and heat shock proteins, which are only released by necrotic tumor cells, acting as a stimulatory and/or maturation signal for infiltrating macrophages and dendritic cells. On the contrary, apoptotic bodies shed by tumor cells undergoing apoptosis have a superior ability to produce CD8+ cytotoxic T-lymphocytes and activate CTLs, T-helper cells, and effector cells of the innate immune system when fed to dendritic cells, and they also act as effective tumor cell vaccines and antigen-presenting-cell-based tumor vaccines. It has also been demonstrated that both types of cell death are necessary for the best cross-presentation of tumor antigens by dendritic cells: a “danger signal” is necessary for the initiation of immunity as presented by necrotic cells serving as a maturation signal, and the phagocytosis of apoptotic cells delivers antigens for presentation by dendritic cells [19]. Further, the PDT-induced release of several immuno-stimulatory molecules, called damage-associated molecular patterns (DAMPs), such as ATP, calreticulin high-mobility group Box 1 (HMGB1), heat shock proteins 70 and 90, and cytokines/chemokines promotes the potentiation of innate and adaptive immunity, constituting an important factor in the long-term development of anticancer immunity and effective tumor control. CD8+ T cells are crucial in the induction of antitumor effects. For instance, after PDT with Photofrin, CD8+ T cells were required to prevent distant lung metastases in a murine EMT6 breast tumor model [20]. Mroz et al. discovered that PDT using verteporfin increased CD8+ T-cell infiltration in distant CT26.CL25 colorectal tumors [21]. Similarly, as discussed above, CD8+ T-cells are involved in cytotoxic effector function resulting in the slow growth of distant tumors in mice with highly aggressive AE17-OVA+ mesotheliomas [22]. As a result, as seen in Figure 1, the optimum PDT regimen necessitates immune system activation in order to combat any cancer cells that may still be present, including distant metastatic cells, and to create a strong immunological memory. Additionally, these post-treatment immunogenic responses can constitute the underlying mechanism of imparting PDT-induced abscopal responses, which have somehow been neglected over the years. Similarly, the generation of certain reactive nitrogen species (RNS) (Table 1) and several immuno- or non-immuno-stimulatory molecules has been investigated with respect to PDT-stress induced bystander effects. Additionally, several other factors may also be responsible for influencing the bystander or abscopal responses in PDT, including the type and concentration of photosensitizer used, the wavelength and intensity of light used, and the microenvironment of the tumor [2,4,23] (Table 1).

**Table 1:** Various kinds of ROS/RNS produced by various PSs have been summarized below.

Various Kind of ROS/RNS	Biological Activity
Superoxide anion (O_2_^•−^), hydroperoxide radical (HOO^•^), peroxides (H_2_O_2_, ROOH) and hydroxyl radical (^•^OH), singlet oxygen (^1^O_2_), nitric oxide(^•^NO)	Mitotic catastrophe (MC), paraptosis, pyroptosis, parthanatos [17], necroptosis, and ferroptosis [16,18], cell signaling, oxidative stress, and immune response [2,4,23]

For PDT, three PS generations have been introduced. Hematoporphyrin derivatives (HpD) were originally introduced by Thomas Dougherty et al. in the 1970s and by Von Tappeiner et al. in 1904 [24,25]. HpD is a chemical compound containing various agents, such as monomers, dimers, and oligomers. The second generation of PSs introduced in the early 1980s comprises synthetic PSs, which have been implemented in some clinical trials. Chlorin, 5-aminolevulinic acid, thiopurine derivatives, texaphyrins, and benzoporphyrin derivatives are the most well-known PSs in this group. These PSs are more efficient than the first-generation PSs and they have some other advantages over this generation, such as high purity and high singlet oxygenation production, but they have poor solubility in water [26,27]. Chemical changes were made to PSs in the third generation to achieve better targeted distribution while lowering phototoxicity to healthy tissues. Second-generation PSs were changed through conjugation with targeting ligands (such as antibodies, carbohydrates, amino acids, and peptides) and encapsulation in carriers (liposomes, micelles, and nanoparticles) to create third-generation PSs. The key goals in the synthesis of the third generation of PSs are as follows: reducing side effects, improving pharmacokinetics, increasing selective and high tumoral accumulation of these PS, and improving PDT effectiveness. The three PS generations are summarized in Table 2 [28]. 

## 3. Biology of Bystander Response in PDT

The bystander effect was first observed in the 1970s when researchers noticed that the irradiation of a small portion of a tumor with ionizing radiation could cause the regression of the entire tumor. It was later discovered that this effect was due to the release of cytokines and other signaling molecules from the irradiated cells, which could stimulate an immune response against the tumor [29,30,31,32,33]. 

In PDT, the bystander effect is the process whereby an injury inflicted on targeted cells may spread to unaffected or unexposed bystander cells (via PSs, light, or both), thereby aggravating treatment-induced tumor damage. Although the bystander effect can enhance the overall efficacy of PDT, it can also lead to damage to healthy tissues surrounding the treated area [3,34,35,36]. The bystander effect in PDT has been observed in various types of cancer cells, including prostate cancer cells [4], breast cancer cells, and glioblastoma [3]. In general, the excellent benefits of PDT include its non-invasiveness, selectivity, propensity to facilitate repeated treatment without the induction of cancer cell resistance, and less severe side effects. However, like any other treatment, an important factor in the outcome of PDT is the treatment response of both normal peripheral cells and surviving cells in the target cancer tissue. While the bystander effect of PDT has both potential benefits and risks, ongoing research is focused on optimizing this therapy to maximize its effectiveness while minimizing its side effects [37,38,39]. The bystander effect occurs through two routes: through the diffusion of molecules in media in cellular gap junctions and the activation of some signals [40,41,42]. However, it is important to note that PDT can also induce bystander effects in normal tissue. The release of cytokines and other signaling molecules from a treated tumor can also affect nearby healthy cells, leading to inflammation and potential damage [43,44]. For instance, burning, stinging, or prickling sensations [45]; hyperpigmentation [46]; hypopigmentation [47]; and urticarial reactions [48] have been reported to be direct or indirect side effects of PDT. Several approaches, such as focal photodynamic injury [35,49,50], co-culturing with non-exposed cells [51], transwell cell culture techniques [5], and the separation of irradiated from unirradiated cells using conical flexi-Perm-ConA silicone rings [34], have been used as models to study bystander signaling in order to understand the underlying mechanism or molecules responsible for inducing the pro- or antitumor effects of PDT-induced bystander effects, which we will discuss in this section.

### 3.1. Contact-Dependent Bystander Effects

Several studies concerning the unraveling of the molecular pathways underlying these treatment-induced effects have shown that the principal mechanism underlying the bystander effect entails direct physical contact between treated and untreated cells by means of gap junctional cell–cell communication involving ions like calcium and small molecules like NO [6,52]. Dąbrowska et al. showed that the co-incubation of healthy cells with PDT-induced necrotic cells resulted in bystander effects, which, in turn, resulted in growth arrest and the loss of cellular adhesion in unirradiated cells due to alterations in focal adhesion kinase gene expression [51]. Confluent monolayers of MDCK II cells exposed to PDT using Photofrin, 3-THPP, TPPS4, and ALA demonstrated an increase in the fraction of dying cells when their dead neighbors were present. They appeared as clusters of dead cells, which was anticipated due to the lipid-peroxidation-chain-reaction-mediated bystander effect [53,54]. Liu et al. showed that gap junctions increase the effectiveness of PDT on cancer cells. They showed that 2-(1-hexyloxyethyl)-2-devinyl pyropheophorbide (HPPH) induced the “death signal” via gap-junction-mediated intercellular diffusion, resulting in an increase in the accumulation of ROS production, which, in turn, potentiated the loss of mitochondrial membrane potential, the activation of caspase-3, and the pathway leading to apoptotic cell death [55]. 

An in vivo study involving a human retinoblastoma xenograft murine model revealed a bystander-effect-mediated propagation of cellular death in the analyzed tumor mass, which was triggered by an initial photoreaction upon the irradiation of 5,10,15-Tri{para-O-[2-(2-O-α-d-manosyloxy)-ethoxy 5353-ethoxy-phenyl}-20-phenyl porphyrin. The results suggested that PDT-induced necrotic cells caused apoptosis in the neighboring nonexposed cells via cell-to-cell death signaling. After two hours of PDT, the onset of tumoral necrosis beneath the skin (as a direct effect) was observed. The emergence of this necrotic region was followed by the appearance of an interfacial zone (i.e., a transitional area between necrotic and undamaged cells), where apoptotic cells were observed. Later, at 48 h, the damage was further propagated into deeper tissue layers, wherein large populations of apoptotic cells were observed at far deeper regions in the tissue, even beyond the light penetration depth [56].

Calcium ion (Ca^2+^) is a key signaling molecule in many cellular processes, including apoptosis and necrosis and in both intracellular and intercellular communication. Depending on the intracellular target of the PS, the cell type, the degree of oxidative stress, and the experimental conditions, PDT-induced ROS generation causes an increase in the intracellular concentration of Ca^2+^ in directly irradiated cells. This increase is caused by Ca^2+^ entry or Ca^2+^ release from the internal stores. The change in the intracellular concentration of Ca^2+^ leads to the activation of downstream signaling pathways that promote cell death either via apoptosis or necrosis depending on the extent of PDT-mediated damage. Interestingly, Ca^2+^ signals have been shown to propagate to surrounding cells as intercellular Ca^2+^ waves via gap junctional communications [57]. Additionally, Ca^2+^ signaling has been linked to the control of PDT-induced immune responses, wherein it modulates the production of cytokines and chemokines immune cells, which can influence the overall activation and recruitment of immune cells at the PDT treatment site [58,59,60]. 

**Figure 2:**
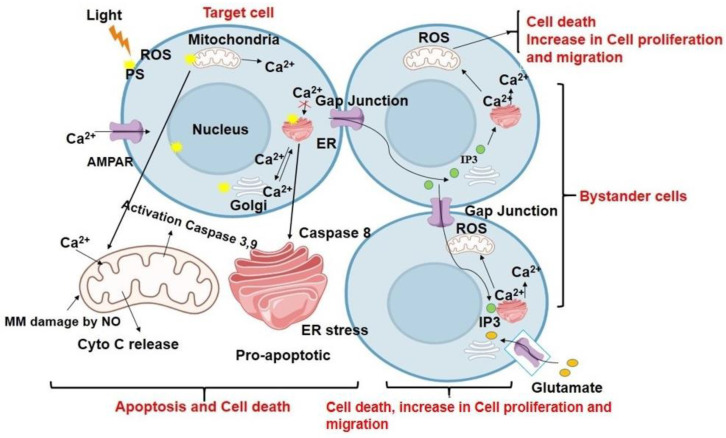
Function of Ca^2+^ in both targeted and untargeted cells. Impact of photodynamic therapy on intracellular Calcium ion (Ca^2+^) concentration required to execute apoptosis and Ca^2+^ function during the process of apoptosis in an irradiated cell. Depending on the intracellular localization of the photosensitizer, light irradiation causes either an increase in the entry of extracellular Ca^2+^ or a decrease in the amount of Ca^2+^ that is taken up by the ER from the cytosol as well as an increase in Ca^2+^ release from the mitochondria and endoplasmic reticulum. Bystander effect caused by photodynamic therapy is mediated by cell-to-cell transmission of Ca^2+^ changes facilitated by Ca^2+^ propagation wave and intercellular gap junctions. This results in propagation and de novo generation of reactive oxygen and nitrogen species, thus inducing the bystander response in the neighboring unirradiated cells.

Studies have shown that Ca^2+^ signaling is also involved in the induction of bystander effects in PDT. The research conducted by Feine et al. showed that a key pathway for the spread of a localized oxidative insult is gap junction intercellular communication between endothelial cells following bacteriochlorophyll WST11- PDT. Follow-up PDT on a small group of cells via a spatially confined oxidative insult resulted in a primarily localized burst of ROS and RNS, generating an intercellular signal, which regulated the spread of damage from the site of injury to distant sites by encouraging bystander cells to produce de novo ROS and RNS. It is interesting to note that while the PDT-targeted cells underwent necrosis, the bystander cells experienced cytochrome-c-dependent apoptosis as a result of the de novo production of ROS and RNS. Furthermore, it was demonstrated that the bystander cells’ apoptotic wave was correlated with ROS-induced activation and the nuclear translocation of c-Jun N-terminal kinase. However, the short half-life and the diffusion limits of ROS exclude the possibility of their propagation via gap junctions to mediate bystander cell death effects. Thus, the observed sharp transient increase in intracellular Ca^2+^ levels suggested that the bystander process can be mediated by Ca^2+^ mobilization from one cell to another through gap junctions [49]. Another study further showed that the photoactivation of AlClPc in a single cell can act as a focal photodynamic injury model and initiate a radial Ca^2+^ wave from the irradiated cell to neighboring bystander cells via gap junctions. The PDT-stress-induced disruption of Ca^2+^ homeostasis results in the generation of NO, which diffuses to nearby cells and raises NO levels further via the Ca^2+^-dependent enzymatic activation of NO synthases (NOS). This radial propagation of a bystander response from the irradiated cell was shown to induce cytochrome-c-release-mediated apoptotic cell death in the bystander cells [35]. This group further extended their study to investigate intraorganellar Ca^2+^ signaling, H_2_O_2_ kinetics in bystander cells, and the dependence of ROS production and mitochondrial Ca^2+^ uptake on the ER-induced Ca^2+^ efflux caused by PS activation. The presently discussed study showed that focal excitation of the PS triggered cytosolic Ca^2+^ release from the ER in both directly irradiated cells and in the bystander cells up to a distance of about ~80 µm. Further, a second generation of ROS driven by mitochondrial Ca^2+^ was induced by ER–mitochondria communication in bystander cells. This was further responsible for the mitochondrial permeability loss resulting in the activation of intrinsic-apoptotic-pathway-mediated cell death in the irradiated and bystander cells [36]. In Figure 2, we have summarized the mechanism of action of Ca^2+^ signaling in bystander effects and cell death after PDT based on the reported studies. 

### 3.2. Diffusing-Mediator-Mediated Bystander Effects

#### 3.2.1. Cytokines

Cytokines are essential elements of the signaling of the innate immune response and can be responsible for the bystander effect. They use autocrine or paracrine processes to exert their effects. Light at high intensity leads to lethal damage and necrosis in tumor and nearby cells via the release of ROS, cytokines, chemokines, and toxic chemicals in mitochondria. Cytokines are signaling molecules that play important roles in the immune system and inflammation [61,62]. Several studies have shown that cytokines such as interleukin-6 (IL-6), tumor necrosis factor-alpha (TNF-alpha), and interleukin-1 beta (IL-1beta) released from PDT-treated cells can induce cell death in neighboring cells and that this process is induced by local and systemic inflammation [63,64]. Dahle and colleagues found that cell–cell contact was not necessary for the mediation of the bystander effect in nearby cells after the PDT treatment of cells in an in vitro culture [53,65,66,67]. Using the WTK1 human lymphoblastoid cell line in suspension and a transwell insert system that prevents contact between the targeted and bystander cells, Chakraborty et al. also demonstrated similar outcomes. These results suggested that rather than just the simple release of intracellular contents from dying cells, induced cell stress and active signaling play a crucial role in inducing bystander responses. This was supported by the findings that lower fluence and a higher surviving population result in bystander effects induced by PDT stress that are more severe in terms of toxicity, DNA damage, mutation fraction, and elevated oxidative stress [5]. The bystander effect of PDT has been demonstrated in various in vitro and in vivo studies, and it has been suggested that it may enhance the therapeutic efficacy of PDT by extending its effects beyond the treated area. An in vivo study by Tseng et al. illustrated that ROS causes the overexpression of p53 and transactivates redox-active proteins, resulting in oxidative-stress-mediated apoptotic cell death in non-target tissues. They used a copolymer of the bPEI_25K_/DNA complex or plasmid DNAs of p53 and KillerRed as a plasmid photosensitizer in PDT. These results suggested the possible role of ROS and the p53-dependent bystander effects of photooxidation in neighboring cells [68]. Furthermore, the potential bystander effect induced by ^68^Ga fibroblast activation protein (FAP) or FAP in combination with PDT on the viability and phenotype of neighboring macrophages was evaluated by Dorst and co-workers. Their findings showed that cell damage and death were upregulated in rheumatoid arthritis synovial biopsies following FAP-tPDT [69]. Moreover, in another study, Anna Dabrowska and colleagues, showed that PDT causes a reduction in mitotic activity and the expression of the focal adhesion kinase gene (FAK) in human ovary cancer cells (OVP10) via a bystander effect. Their data showed that the density decreased significantly, i.e., by 21–28%, after 24 and 48 h in cells co-cultured with PDT. In addition, bystander growth arrest was attributed to a significant decrease in mitotic activity at 24 h and a lower expression of FAK [51]. Further, Dahle et al. reported an interesting phenomenon consisting of a stronger bystander effect for cancer cells killed by necrosis than that for cells undergoing apoptosis at doses inactivating more than 50% of the cancer cells treated with 3-THPP and irradiated. This finding was in accordance with the hypothesis of a bystander effect mediated by signals released into a medium; thus, cells undergoing necrosis release an increasing number of toxic substances, which can easily diffuse into the microenvironment around a cancer colony and may eventually kill the neighboring cells [66] (Table 3). 

**Table 3:** Photodynamic-therapy-induced bystander response in in vitro and in vivo studies.

Types of PS or Other Agent	Cell Lines/Tissue	In Vitro/In Vivo	Effect or Mechanism (Molecular Response)
HPPH	EMT6	In vivo	Levels of Macrophage inflammatory protein (MIP) and IL-6 increased [64]
bPEI_25K_/DNA-complex	H1299	In vitro/in vivo	Overexpression of p53 [68]
^68^Ga-FAP	synovial tissue	ex vivo	Overexpression of caspase-3 [69]
Hematoporphyrin derivative (HpD-Arg(2)	OVP10	In vitro	Reduced mitotic activity and expression of the FAK [51]
Tetra(3-hydroxyphenyl)porphyrin	MDCK II	In vitro	Increased necrosis [66]
Deuteroporphyrin (DP)	WTK1	In vitro	DNA damage increased [5]

These studies suggested several different possible mechanisms for the bystander effect mediated by diffusible molecules. The authors neglected the involvement of the PDT-induced generation of singlet oxygen as a mediator or initiator of bystander effects because singlet oxygen’s brief lifetime in cells renders extranuclear formation, diffusion, and subsequent reactions difficult. Therefore, it is more likely that primary singlet oxygen will cause membrane damage and that secondary mediators will be produced, which can diffuse both inside and outside of cells to initiate oxidative damage. Other likely mechanisms involved in triggering oxidative bystander effects include PDT-induced membrane damage and lipid peroxidation. This process was proposed because ROS, like hydrogen peroxide and other byproducts of lipid hydroperoxides, have lifetimes that are long enough for them to diffuse into nearby cells [5,66].

#### 3.2.2. Oxidative Species

Other than singlet oxygen and free radicals, H_2_O_2_ can also be a potential mediator of bystander responses due to its long lifetime and uncharged nature, allowing it to diffuse extracellularly in a medium and thus into distant cells. In this context, a few studies have shown that the oxidative stress induced by the PDT of a targeted cell subpopulation can spread to the untreated cell population via bystander signaling. This was suggested to occur due to the photodynamic-stress-mediated activation of NADPH–oxidase in the targeted cells, resulting in a rapid burst of a wave-like signaling process with H_2_O_2_ production. This process can initiate PDT-induced bystander responses and an overall PDT assault in a cell population [50,70]. As discussed in the previous section, the bystander effect induced by PDT is frequently attributed to Ca^2+^ signaling via IP3- and ATP-based propagation mechanisms, which are thought to be involved in the de novo regeneration of NO and/or ROS in bystander cells. Thus, this process results in the generation and further propagation of bystander cells via ROS (majorly H_2_O_2_) and/or NO, which generally have limited diffusion distances as signaling mediators [57]. NO is a bioactive free radical molecule with a short lifetime of 1–2 s in an aqueous environment and can easily diffuse freely. The relative stability and hydrophobic characteristics of NO allow for its diffusion through the cytoplasm and plasma membranes over several cell diameter distances, thereby contributing to its distinctiveness as a redox-signaling molecule in bystander responses that does not require gap junctional communications. NO is produced by a group of enzymes called NO synthases, whose actions determine whether it exhibits pro-tumor vs. antitumor properties depending on the extent of its steady state levels. It plays a critical signaling role in the survival, proliferation, migration, and drug resistance of cancer cells at low to moderate steady state levels, namely, between 50 and 500 nM, while inducing cytotoxicity in macrophages at relatively high levels, namely, above 1 µM [71,72]. Research has shown that PDT can be compromised in several ways by stress-induced iNOS, which causes NO-induced hyper-resistance and aggressiveness [3,34,73]. According to the proposed explanations of several studies, PDT’s vasoconstrictive effects are counteracted by vasodilation in tumor blood vessels mediated by NO signaling [74]. Subsequently conducted studies revealed that photodynamic stress itself upregulated iNOS mRNA and protein levels in PDT-treated cells, increasing resistance to apoptotic photokilling, enhancing migration and invasion by activating matrix metalloproteinase-9 (MMP-9), and inhibiting MMP-9 TIMP-1 [75,76,77]. The following sequence of events was suggested: Akt was activated by photo stress and thus NF-kB was activated, resulting in the upregulation of iNOS transcription/translation and NO production, leading to apoptosis resistance [3]. The antitumoral response to PDT can be influenced by both endogenous NO and exogenous NO produced by a photoactivated PS, constituting an interesting revelation [78]. Photooxidative-stress-induced NO is crucial in determining the course of a PDT-treated tumor and the overall treatment outcome [78,79]. Low NO levels are associated with low-dose-PDT-activated molecular survival pathways such as caspase inactivation via S-nitrosylation protein kinase G activation and the suppression of pro-apoptotic JNK and p38 MAPK pathways, resulting in the growth of populations of aggressive and resistant tumors. Additionally, because of its antioxidant effects and activation of the antioxidant Nrf2, pro-survival NF-kB, and KRAS/MEK signaling pathways, NO plays a cytoprotective role at low doses, thus reducing the ROS levels induced by PDT [4,79,80]. On the other hand, tumor growth is retarded by high-dose PDT combined with high levels of NO. This complex behavior of NO was excellently demonstrated in the study conducted by D’Este et al., wherein two photo-oxidative treatment setups were used. The first model involved repeated low-dose Pheophorbide-a PDT to generate a low chronic level of oxidative stress in cancer cells, which led to the development of a more aggressive subpopulation with the following characteristics: (i) the CD44+/CD24+ phenotype; (ii) the ability to produce tumorspheres; (iii) the increased expression of various “stemness” genes like iNOS, HIF-1, SOX-2, and NANOG; (iv) and the epithelial-to-mesenchymal transition, resulting in the tumor cells developing invasive and migratory characteristics. While in the second model, a single high dose of Pheophorbide-a PDT generated high levels of iNOS/^•^NO which resulted in oxidative stress induced cell growth arrest. Further, their study showed that ^•^NO controls the molecular pathways activated in response to oxidative stress by acting as a “redox switch”. NO regulates the NF-κB/YY1/Snail/RKIP loop, which involves both anti-apoptotic and pro-survival modulators [81,82]. As discussed in Section 3.1, PDT elicits a sharp rise in intracellular Ca^2+^ levels, resulting in the propagation of a Ca^2+^ wave originating from the targeted cell to bystander cells up to a certain distance, with the simultaneous increase in the production and release of NO from both irradiated and non-irradiated cells. Subsequent to these events, the release of ions, cytokines, and other secondary messengers transmits a signal to the intercellular signaling network that leads to the bystander effect in tumors [57]. 

**Figure 3:**
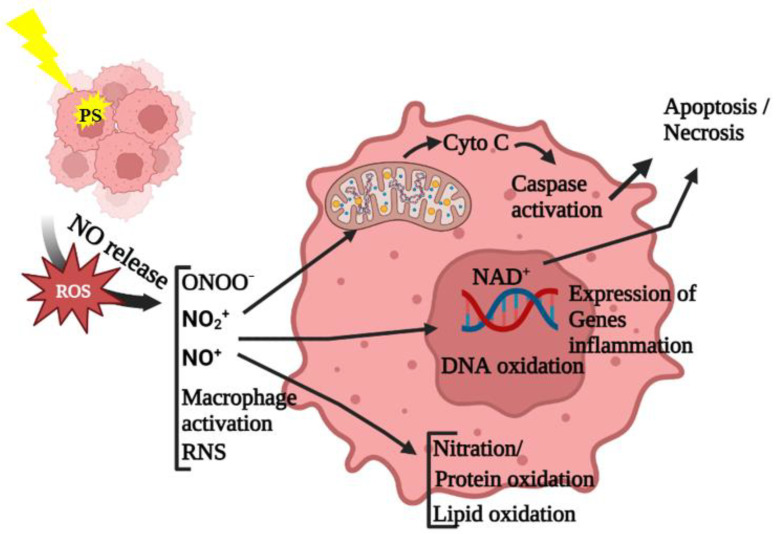
Schematic representation of photo-oxidative-stress-dependent stimulation of cell death in targeted and untargeted cells. Target cells that have undergone photodynamic therapy release reactive oxygen and nitrogen species, resulting in a chain reaction of oxidative species generation. This buildup of oxidative stress ultimately results in both target and neighboring bystander cell death via damage to cellular organelles and macromolecules.

Bazak et al. investigated the bystander effects of ALA-PDT on various cell lines, where the targeted and untargeted cells were segregated into two cell populations via impermeable silicone-rimmed rings. The results showed that a uniform, moderate level of targeted PDT cell killing resulted in an enhancement in both the proliferation and migration mediated by bystander effects of diffusible NO. This was highly correlated with the upregulation of iNOS/NO in both target and bystander cells. Further, the NO-mediated bystander effect was associated with a potent but brief activation of the Akt and ERK1/2 kinases as well as the induction of cyclooxygenase-2 (COX-2), which acted as potential pro-survival/pro-growth effector proteins. This resulted in the more aggressive growth and migration of cancer cells post-irradiation [34]. In an interesting study, it was shown that cells’ level of malignancy determines whether mild photo-oxidative stress mediated by NO results in a bystander effect. The results showed that among all the prostate cancer cells, only PC3 prostate cancer cells that were more resistant and malignant than other types of cells responded to the bystander effect mediated by conditioned media from cells treated with low-level PDT, resulting in an increase in iNOS and S-nitrosoglutathione reductase in PC3 bystander cells. Furthermore, the researchers demonstrated and hypothesized that NO only has a “remote” bystander effect in androgen-unresponsive cancer cells. This finding could be explained by the difference in cell morphology between fibroblasts and epithelial cells (epithelial cells express more vimentin) as well as the possibility that GSH and S-nitrosoglutathione reductase regulated the balance between nitrosylation and denitrosylation [4]. Figure 3 summarizes the role of NO in modulating cell death in the target and nearby cells.

## 4. Biology of Abscopal Response in PDT

The word “abscopal” was first used in 1953 by R. H. Mole to describe an action apart from the irradiated volume but within the same organism. The abscopal effect has been recorded among patients with metastatic cancer treated with radiation alone despite it being a rare or variable occurrence and poorly understood. There were 46 case reports published between 1969 and 2014 regarding multiple different primary tumors, ranging from melanoma to cholangiocarcinoma and renal cell carcinoma, showing non-irradiated, distant responses, usually occurring two months after radiation. This effect is only well studied with respect to radiotherapy [83,84,85,86]. The abscopal effect in PDT was first noted in the 1990s when researchers noticed that, in animal models, treating a single tumor with PDT could cause distant tumors to regress [87]. In one phase II clinical trial, patients who underwent Photofrin-PDT for intraperitoneal tumors primarily originating from ovarian cancer 2 days before tumor-debulking surgery responded well to the treatment, with a median survival of 21 months [88]. However, the first clinical case report was reported in 2007, wherein, following PDT, the regression of distant tumors that had not been treated was observed. The patient, who presented histologically proven multifocal angiosarcoma of the head and neck and a relapsed tumor following high-dose brachytherapy, was treated with a next-generation PS, Fotolon, which has a 1:1 ratio of polyvinylpyrrolidone and chlorin e6. In this report, a biopsy of the untreated tumor showed the activation and proliferation of peptide-specific cytotoxic CD8+ T-cell clones and a shift from CD4+ to CD8+ T-cell infiltration [89].

The theranostic PS PLP was shown by Zheng’s group to be effective in treating a metastatic VX2 buccal carcinoma rabbit model. Interestingly, they reported that despite the fact that the site had not been laser-irradiated, the elimination of the VX2 primary tumor occurred concurrently with the regression of lymph node metastasis. The abscopal effect, in which the treatment of a primary tumor can cause a systemic immune response that can control or eradicate secondary, untreated tumors, was raised as an interesting possibility by this study, with the potential use of PLP-mediated PDT to achieve the effect [90]. In another study, the application of a nano-emulsion containing aluminum-phthalocyanine followed by PDT treatment was shown to be successful in eliminating 4T1 breast adenocarcinoma tumors in mice and in preventing the development of lung metastasis [91]. 

It has been reported that the release of DAMPs, which mainly occurs following local therapies, is required to induce the abscopal effect [86]. The production of the proteins calreticulin and high-mobility group box-1 (HMGB1), two main representatives of the DAMPs group, following PDT-induced cell damage is responsible for the activation of the immune response in the form of an antitumor host response induced by PDT [92]. However, the role of the expression of the calreticulin chaperone protein on the cell surface in response to PDT-induced ER stress as an indicator of immunogenic cell death and the induction of the abscopal effect is still unconfirmed. For example, according to a few studies, treatment consisting of PLP loaded into Zn-pyrophosphate (ZnP) nanoparticles and coordination polymer core–shell nanoparticles followed by PDT demonstrated increases in the expression of calreticulin in breast and colorectal tumor models [93,94]. In a similar study, the talaporfin-sodium-PDT-induced release of several DAMPs, such as DAMPs in ICD including calreticulin, heat-shock protein, ATP, and high-mobility group protein B1, was correlated with the strengthening of the abscopal effect on the non-irradiated side in a syngeneic colon adenocarcinoma mouse model of bilateral flank tumors [95]. A translocator protein-targeted PS IR700DX-6T also showed a direct and abscopal effect in a syngeneic immunocompetent colorectal mouse model post-PDT, whereby the increased expression of calreticulin and the release of HSP70 resulted in the activation of a host antitumor immune response mediated by the activation of dendritic and CD8+ T cells along with a decrease in the Treg cell population in both treated and non-treated tumors [96]. However, the highly aggressive dual subcutaneous AE17-OVA+ mesothelioma mouse model used in the study by Lou et al. presented a reduction in the expression of calreticulin in PDT-treated tumors. Importantly, repeated cycles of PDT are required to delay the growth of distant nonirradiated tumors [22,94]. 

An investigation of the underlying immune mechanism in inducing abscopal effects revealed high serum interleukin-6 levels, suggesting the activation of the innate immune system, which probably helped to attract neutrophils, dendritic cells, and macrophages. Additionally, it was reported that non-irradiated tumors had higher percentages of CD4+ T cells and effector memory CD8+ T cells and lower percentages of central memory CD4+ T cells in the spleen. Then, dendritic cells primed undifferentiated CD8+ T cells, inducing their differentiation into effector CD8+ T cells and, subsequently, effector memory phenotype cells. In addition, CD4+ T cells support CD8+ T cells in establishing and maintaining CD8+ effector memory. Granzyme A, Granzyme B, perforin, the fas ligand, trail, and IFN were upregulated as a result of these CD8+ effector memory T cells migrating to a distal tumor, which caused cytotoxic effects and inhibited the growth of the distal tumor [22]. Further, this group conducted experiments to investigate the broader immune mechanisms involved in monotherapy PDT and its combination with immunotherapy in the induction of abscopal effects. C57BL/6 mice with subcutaneous AE17-OVA mesothelioma dual tumors were subjected to three different treatment groups consisting of anti-PD-1 monoclonal antibody, repeated PDT, and combination therapy. Repeated PDT and combination therapy showed that broad innate immune activation mediated a substantial increase in interleukin 6. Due to increased dendritic cell and macrophage expression of MHC class II, CD80, and CD86, the spleen and distal, non-irradiated tumor-draining lymph nodes had a higher propensity for antigen presentation in the same treatment groups. In addition, the proportion of CD8+ T cells increased in the distal, non- irradiated lymph nodes that drain the tumor while simultaneously changing CD4+ T cell ratios in the spleen. However, monotherapy PDT showed a promising safety profile compared to combination therapy, which induced mild tumor lysis syndrome [97]. 

**Figure 4:**
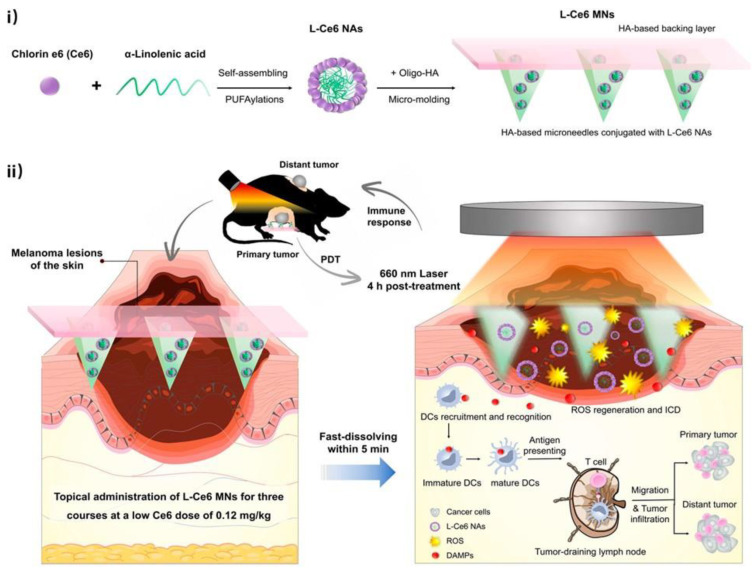
(**i**) Schematic illustration of fabrication of L-Ce6 microneedles; (**ii**) L-Ce6 microneedles effectively delivered L-Ce6 nano-assemblies into tumor lesions and ablated the primary tumor by producing ROS following 660 nm laser irradiation. L-Ce6 MNs then induced ICD and released DAMPs to activate antitumor immune responses, increasing T cell infiltration in both bilateral tumors and repressing distant tumor growth. Adapted with permission from Bian et al. [99], Copyright 2021 American Chemical Society.

Similarly, Ce6-PDT was also reported to induce potent local and systemic antitumor immune responses in syngeneic B16F10 melanoma and Panc02 pancreatic tumor mouse models. The antitumor and abscopal effects of Ce6-PDT were associated with PD-1/PD-L1 interaction inhibition correlated with an enhanced frequency of CD8+ T cells, increased Granzyme B levels, reduced CD39+ T cell activity, and elevated IL-2 release [98]. In another study, Photofrin PDT on subcutaneous tumors of EMT6 tumor cell murine models bearing both primary and lung tumors showed CD8+-T-cell-dependent inhibition of the growth of untreated lung tumors. In this regard, increased splenic antitumor cytolytic activity and CD8+ T cell infiltration into untreated tumors were suggested to be responsible for this inhibition [20]. In an interesting study, carrier-free L-Ce6 nano-assemblies—integrating a rapidly dissolving microneedle patch—were developed to achieve precise and effective drug delivery to tumor lesions. The L-Ce6 MNs-based PDT effectively generated ROS to ablate the primary lesions in situ as well as distant lesions in a B16F10 melanoma xenograft model, even at a low drug dose of L-Ce6. More importantly, the low L-Ce6 dose was observed to boost tumor immunogenicity by inducing a significant abscopal effect via triggering immunogenic cell death (ICD), releasing danger-associated molecular patterns, and promoting dendritic cells’ maturation and subsequent antigen presentation, thereby aiding the T-cell-mediated immune response without the need for synergistic immunotherapies (Figure 4) [99]. 

According to some studies, PDT frequently fails to produce a strong abscopal effect on its own. Thus, approaches based on combining photodynamic and immunological therapy to enhance the abscopal effect have also gained interest. The combination of PDT with immunotherapies has been proposed in order to improve antitumor efficacy toward primary irradiated tumors and promote systemic immune responses against metastases because of the various mechanisms of cell death. In order to prevent relapses and help patients to achieve long-term remission, PDT and immunotherapies may be combined. The immune memory that is created will ensure these goals are reached by preventing relapses and assisting patients in achieving long-term remission [95,97]. 

**Figure 5:**
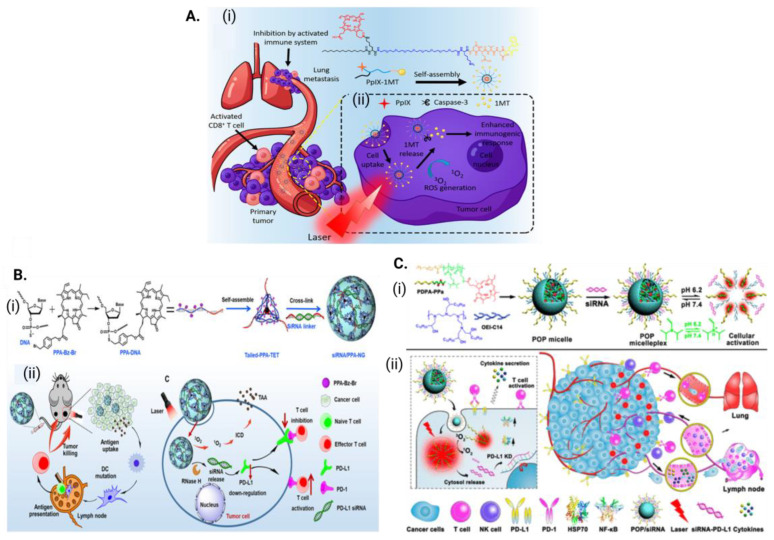
Schematic illustration of structure of (**A**) chimeric peptide PpIX-1MTB nanoparticles (adapted with permission from Song et al. [100]; Copyright 2018 AMERICAN CHEMICAL SOCIETY). Programmed assembly of (**B**) siRNA/PPA-NG (adapted with permission from Guo et al. [102], 2022 American Association for the Advancement of Science). (**C**) Acid-activatable micelleplexes (adapted with permission from Wang et al. [101]; Copyright 2016 AMERICAN CHEMICAL SOCIETY) and their synergistic functions in cancer photoimmunotherapy.

Several studies (shown in Table 4) based on immune-stimulating nanoparticles loaded with PSs as a PDT strategy in combination with an immune checkpoint blockade have shown that the corresponding combination treatments can effectively destroy primary tumors when exposed to light, inhibit distant tumors through abscopal effects that are otherwise difficult for light to reach, and prevent tumor recurrence via the immune memory effect. In an effort to combine PDT and immunotherapy in a single structure, Song et al. developed a chimeric peptide, PpIX-1MT, incorporating the photosensitizer Protoporphyrin IX and 1-methyltryptophan as an immune checkpoint inhibitor. The PpIX-1MT nanoparticles accumulated effectively in tumors through the EPR effect, which, upon the tumor cells’ irradiation with 630 nm light, resulted in the ROS-induced apoptosis of tumor cells (Figure 5A). The activation of the apoptotic pathway resulted in the production of caspase-3, which then allowed for the release of 1MT, which activated an immune response that efficiently recruited more DCs and CD8+ T cells. Thus, the primary tumors and lung metastasis tumors in CT26 colorectal cancer murine models were effectively inhibited using this cascaded synergistic effect [100]. Some studies have shown that using RNA interference (RNAi) to genetically alter the PD-1-PD-L1 pathway can also improve the ability of PDT-induced cancer immunotherapy to induce abscopal effects (Figure 5B,C). For example, pheophorbide a coloaded with PD-L1 siRNA in a nucleic acid nanogel and acid-activatable versatile micelleplex demonstrated significantly improved effectiveness with respect to preventing tumor growth and distant metastasis [101,102].

## 5. Challenges

The significant obstacles to PDT’s ability to generate effective bystander and abscopal effects and long-lasting systemic immunity against cancer include the following: (1) the short life time of ROS produced after PDT are possibly ineffective in treating tumors, and the offensive production of DAMPs is triggered by ineffective cell stress, which plays a crucial role in eliciting an immune response, and (2) hypoxic tumor conditions or PDT-induced hypoxic conditions reduce the extent of ROS generation, resulting in immunosuppressive effects. 

## 6. Conclusions

Although the discovery of the bystander and abscopal effects induced by PDT dates to 1990s, these crucial phenomena have not been considered until the last decade. The bystander and abscopal effects with respect to PDT are still poorly understood, and their occurrence varies significantly despite these encouraging results. PDT itself is a combination of several different factors and conditions. Thus, the likelihood of these untargeted effects occurring can be influenced by a number of variables, including the type of cancer, the photosensitizer used, its subcellular localization, the timing, the dose of light exposure, the extent of damage, cell death, and the activation of the immune response. 

The literature has shown the crucial potential roles played by both contact-dependent and -independent intercellular communications and the involvement of highly stable and longer-lifetime oxidative radicals, like H_2_O_2_, NO, and Ca^2+^ flux, in inducing strong bystander effects after PDT. Preclinical have studies proposed the involvement of the PDT-induced release of DAMPs and CD8+T cell activation during the abscopal response, which subsequently stimulates the immune system on a broader level to kill treated and untreated tumor masses, thereby contributing to the long-term prevention of cancer recurrence. However, due to the complex interplay of different factors, it is challenging to link the complete response to a single effect or mediator. As a major player, ROS are suggested to be crucial for bystander and abscopal effects, but both the untargeted responses and their underlying signaling machinery communication are unpredictively dependent on the extent of damage such as suboptimal death, cellular death pathways, repeated treatment, and many other forms. 

As discussed, one of the major limitations of PDT-induced bystander and abscopal effects is inherent microenvironment hypoxia and/or PDT-induced hypoxia. In this respect, drug delivery systems are potential agents that can increase the production of ROS, thus regulating the tumor’s hypoxia such that it reaches a normoxic state. Secondly, it is crucial to comprehend the type of immune suppression, i.e., either via immune checkpoint blockade or the tumor microenvironment, so that the proper combination therapy can be applied in order to overcome suppression and avoid immune-related adverse events. Some strategies involve combining PDT with treatments like immune adjuvants, immune checkpoint blockers, indoleamine 2,3-dioxygenase inhibitors, immune adjuvants, HIF1 inhibitors, etc.

Some of the areas that warrant more extensive research are as follows: (a) a thorough investigation of the involvement of T cells and cytokines; (b) appropriate PDT doses; (c) the investigation of the inherent potential or modifications of PSs that will lead to the production of enough ROS over time and thus induce the release and activation of appropriate mediators; (d) and an investigation of all the potential biomarkers of the tumor microenvironment, immune system, and oxidative and nitrosative stress related to the bystander and abscopal responses. 

The effectiveness of targeted and untargeted PDT responses and their use in clinics could both be improved with more research in this area. This will help to mitigate any induced negative effects on the surrounding healthy tissue, reduce the burden of chemotherapy and other combined therapies, and provide a clinical solution to the problem of tumor recurrence and metastasis. 

## Figures and Tables

**Table 2:** Various generations of PS drugs used in PDT have been summarized.

Generations	Family and Name of PS
G1	Porphyrin family: HpD, BPD (benzoporphyrin derivative), ALA, Texaphyrins [15,25].
G2	Chlorin family: Temoporfin, Purlytin(tin-ethyl-etiopurpurin), NPe6(mono-L-aspartyl chlorin e6), LS11(Talaporfin sodium), HPPH (Photochlor), 5-aminolevulinic acid (ALA), Benzoporphyrin derivative (BPD), Tinethyletiopurpurin (SnET2), Bacteriochlorins, Methylene blue derivatives, Toluidine blue, Phthalocynine, Curcumin [26,27].
G3	Dye family: Naphthalocyanine (tin2,3naphthalocyanine), Phthalocyanine [28].

**Table 4:** Various preclinical studies on photodynamic therapy in combination with immunotherapy showing abscopal effects.

PS/PS-Based Nanostructures/Formulations	Immune Checkpoint Inhibitors	In Vivo Model
Upconversion nanoparticles loaded with chlorin e6 (PS) and imiquimod (R837) (Toll-like-receptor-7 agonist)	CTLA-4 *	CT26 colorectal cancer murine model [103]
Cancer-cell-membrane-cloaked Janus magnetic mesoporous organosilica nanoparticles loaded with chlorin e6	CTLA-4	4T1 breast cancer murine model with lung metastases [104]
Phthalocyanine derivative albumin supramolecular complexes	PD-1 * or PD-L1 *	4T1 breast cancer murine model [105]
Supramolecular self-assembly of morpholine-modified silica phthalocyanine (PcM) and serum albumin (SA)	PD-1	4T1 breast cancer murine model with lung metastases [106]
Zn-pyrophosphate (ZnP) nanoparticles loaded with pyrolipid (photosensitizer)	PD-L1	4T1 and TUBO breast cancer murine model with lung metastases [93]
Core–shell nanoparticles with oxaliplatin in the core and the PS pyrolipid in the shell	PD-L1	HT29 and CT26 colorectal cancer murine model [94]
Cancer-associated-fibroblast-targeted FAP-specific single-chain variable-fragment (scFv)-conjugated ferritin nanoparticles loaded with ZnF16Pc	PD-1	4T1 breast cancer murine model [107]

* CTLA-4: Cytotoxic T-lymphocyte-associated protein 4; PD-1: Anti-programmed death protein; PD-L1: Anti-programmed death ligand 1.

## Abbreviation

PDT	Photodynamic therapy
ROS	Reactive oxygen species
PS	Photosensitizer
ICD	Immunogenic cell death
NO	Nitric oxide
MC	Mitotic catastrophe
DAMPs	Damage-associated molecular patterns
RNS	Reactive nitrogen species
ALA	5-aminolevulinic acid
HPPH	2-(1-hexyloxyethyl)-2-devinyl pyropheophorbide
Ca^2+^	Calcium ion
NOS	Nitric oxide synthesis
TNF-α	Tumor necrosis factor-alpha
FAP	Fibroblast activation protein
MIP	Macrophage inflammatory protein
MMP-9	Matrix metalloproteinase-9
COX-2	Cyclooxygenase-2
GSH	Glutathione
PLP	Porphyrin lipoprotein
HMGB1	High-mobility group box-1
ZnP	Zn-pyrophosphate
